# Contextual fear learning and memory differ between stress coping styles in zebrafish

**DOI:** 10.1038/s41598-019-46319-0

**Published:** 2019-07-09

**Authors:** Matthew R. Baker, Ryan Y. Wong

**Affiliations:** 1 Department of Biology, University of Nebraska at Omaha, Omaha, Nebraska USA; 2 Department of Psychology, University of Nebraska at Omaha, Omaha, Nebraska USA

**Keywords:** Behavioural ecology, Fear conditioning

## Abstract

Animals frequently overcome stressors and the ability to learn and recall these salient experiences is essential to an individual’s survival. As part of an animal’s stress coping style, behavioral and physiological responses to stressors are often consistent across contexts and time. However, we are only beginning to understand how cognitive traits can be biased by different coping styles. Here we investigate learning and memory differences in zebrafish (*Danio rerio*) displaying proactive and reactive stress coping styles. We assessed learning rate and memory duration using an associative fear conditioning paradigm that trained zebrafish to associate a context with exposure to a natural olfactory alarm cue. Our results show that both proactive and reactive zebrafish learn and remember this fearful association. However, we note significant interaction effects between stress coping style and cognition. Zebrafish with the reactive stress coping style acquired the fear memory at a significantly faster rate than proactive fish. While both stress coping styles showed equal memory recall one day post-conditioning, reactive zebrafish showed significantly stronger recall of the conditioned context relative to proactive fish four days post-conditioning. Through understanding how stress coping strategies promote biases in processing salient information, we gain insight into mechanisms that can constrain adaptive behavioral responses.

## Introduction

When animals successfully overcome stressors (e.g. predation, resource acquisition), cognitive processes facilitate the encoding and recalling of these salient experiences to modify or reinforce beneficial coping behaviors in the future. Within an individual, behavioral and physiological responses to stress often co-vary as part of a correlated suite of traits that are consistent across contexts and time (i.e. animal personality)^1–4^. Animals that are risk-prone or risk-averse differ in boldness, aggression, and stress physiology, and represent opposite ends of a response continuum observed across many taxa (e.g. bold-shy, proactive-reactive axis)^2–5^. While variation in cognitive abilities can be due to a variety of factors^6–9^, studies are beginning to demonstrate that learning and memory processes are also biased according to personality type^7,8,10–12^.

In line with other behavioral and physiological traits, studies suggest that proactive and reactive stress coping styles differ in information processing, decision making, and learning and memory capabilities^4,7,11–14^. The more risk-prone proactive individuals tend to rely on past experiences and form more rigid routines (i.e. low behavioral flexibility). In contrast, the risk-averse reactive individuals are more sensitive to environmental cues for learned associations and display higher behavioral flexibility. Despite these observations, there are inconsistencies across studies investigating how learning and memory abilities vary with personality type in mammals, birds, and teleosts, often relating to the type of paradigm and stimulus valence. Some studies show that reactive individuals will learn faster^15–17^, but others show support for proactive individuals learning faster^18–24^. The same conflicting observations are documented with memory performance between the stress coping styles^10,16,25^. Examining to what extent encoding and recalling of salient information is influenced by stress coping style is important towards understanding factors that may facilitate the development of correlated suites of traits within an individual.

Exposure to highly stressful events such as predation are useful for investigating individual differences in learning and memory. Upon experiencing a threatening event, an individual can associate a specific cue of the threatening stimulus and the general environment in which it was experienced (e.g. context)^26^. Many learning paradigms utilize predator odors or chemical alarm signals as an unconditioned stimulus (US) to study ecologically relevant cognitive behaviors^27^. In teleosts a chemical alarm signal (alarm substance) is released from epidermal cells when they are mechanically damaged. This olfactory signal causes robust antipredatory behaviors even in the absence of a predator, and is used to assess stress-related behaviors in zebrafish (*Danio rerio*) and other teleosts^28,29^. Typical fear responses in teleost include bottom dwelling, swimming in a tighter shoal, erratic movements and freezing. While studies have utilized alarm substance for associative conditioning paradigms of specific cues on schools of fish, it has presented some challenges for measuring individual differences in learning and memory^10,30–34^. Furthermore, not much is known whether alarm substance can be used for contextual learning and recall of salient information. Utilizing alarm substance to study the relationship between learning, memory, and personality types will require behavioral assays that can be tested on individual fish, are rapidly and reliably acquired, and allow for isolated examination of both learning and memory recall phases.

Here we use zebrafish to study how cognitive abilities vary with stress coping style. Zebrafish are utilized in a variety of laboratory studies to understand the neural, genetic, and pharmacological mechanisms of learning and memory^35–37^. Both wild and laboratory strains of zebrafish display the proactive and reactive stress coping styles, which have distinct genetic architectures and neuroendocrine responses^38–41^. Given their ability to demonstrate learning and memory behaviors, and possess different personality types, zebrafish are a promising system to study how an animal’s stress coping style influences fear learning and memory abilities^28,36,37,42–46^.

The goal of the study was to understand how the animal’s personality influences learning and memory in an associative fear conditioning task. Our study was designed to investigate (1) the hypothesis that two strains selectively bred to display proactive and reactive stress coping styles present distinct profiles to learn that a determined context is potentially dangerous. We also tested (2) if evocation of fear memory conditioned to this specific context is distinct between strains, and (3) the utility of a novel contextual fear conditioning paradigm in assessing individual differences. We predict that if there are differences in ability to acquire a contextual fear association between stress coping styles in zebrafish, we will observe a significant stress coping style*treatment*conditioning trial interaction effect during training for fear-related behaviors (e.g. significantly higher freezing over time in one stress coping style with repeated exposure to alarm substance in training paradigm). Further, if there are differences between stress coping styles in the ability to retain the contextual fear memory, we predict there to be a significant stress coping style*treatment interaction effect for fear-related behaviors during the memory recall trials (e.g. significantly different freezing times between stress coping styles that underwent contextual fear conditioning).

## Methods

### Subjects

We used the high-stationary behavior (HSB) and low-stationary behavior (LSB) zebrafish strains^47^. Starting from wild-caught zebrafish, the HSB and LSB strains were generated and are maintained by artificial selection for opposing amounts of stationary behavior during an open field test in each generation (see ref.^47^ for selective breeding details). The LSB strain show consistently higher risk-prone behaviors across 5 different behavioral assays, larger caudal fin and fast-start escape responses, lower post-stressor cortisol levels, and distinct basal neurotranscriptome profile than the HSB strain^40,41,47–51^. Additionally, these divergent behavioral profiles between the strains are consistent over time and are highly repeatable^52^. Thus, collectively the HSB and LSB strains on average show characteristics consistent with the reactive and proactive coping styles, respectively^4,7,11–14^.

We randomly selected 32 individuals for each of the LSB and HSB strains from their stock tanks and assigned each to one of two groups. Fish that did not display any response to the US (alarm substance) were removed from the study, resulting in a final sample size of 24 LSB (N = 12 males, 12 females) and 24 HSB (N = 12 males, 12 females) for the treatment group receiving alarm substance during conditioning. An additional 8 LSB (N = 4 males, 4 females) and 8 HSB (N = 4 males, 4 females) were used as a control group being exposed to distilled (DI) water during conditioning. LSB and HSB individuals were 16 months post-fertilization when testing began, and were 10 generations removed from a wild caught population from Gaighata in West Bengal, India. While age-related decline in learning and memory performance were found in zebrafish 36–60 months old^53,54^, we used zebrafish at an age where there is no document of age-related decline in cognition. During testing fish were individually housed in 3-liter tanks on a recirculating water system (Pentair Aquatic Eco-Systems) using UV and solid filtration on a 14:10 L/D cycle at a temperature of 27 °C. Prior to testing fish were housed in mixed-sex 40 L tanks on a custom-built recirculating water system with same filtration, light cycle and water temperature. Fish were fed twice a day with Tetramin Tropical Flakes (Tetra, USA).

### Alarm substance

We created a single batch of alarm substance following modified guidelines using 20 randomly selected donor fish^29^. In brief, donor fish were euthanized by rapid chilling followed by light abrasion of lateral skin cells on one side of each donor fish, ensuring that no blood was drawn. Donor bodies were then individually soaked in 10 mL of DI water for 10 minutes. We determined a working concentration through a pilot dose-response study following procedures used in one of the conditioning trials of the contextual fear learning paradigm (see below). In brief, fish were individually placed into an acrylic testing arena (16 × 16 × 10 cm) surrounded by opaque white plastic on the bottom and sides, and filled with 1.4 L of system water. After ten minutes we administered one of four concentrations of alarm substance (0% (DI water), 10%, 50%, 100% alarm substance), and quantified freezing duration for the subsequent five minutes. The 50% concentration elicited a significantly higher increase in freezing behavior compared to the DI water (*t*(22) = 3.24, *p* = 0.004, d = 2.33) and 10% (*t*(22) = 3.15, *p* = 0.005, d = 2.14) alarm substance administrations (Fig. S1). We therefore selected 50% as the working concentration. A total of 200 mL was filtered, diluted in half, and stored in aliquots at −20 °C until use.

### Contextual fear learning

To assess learning and memory we developed a novel contextual fear conditioning paradigm. Zebrafish were tested individually in an acrylic testing arena (16 × 16 × 10 cm) filled with 1.4 L of system water. The arenas were surrounded by opaque white plastic on the bottom and sides to serve as the contextual stimulus. A second distinctly different context consisted of red plastic on the bottom with a picture of underwater plants on the side walls served as a control.

**Figure 1:**
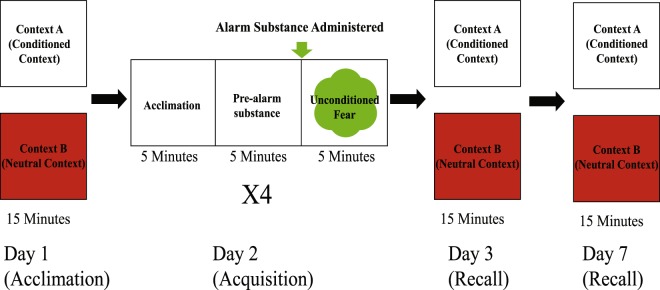
Contextual fear conditioning protocol. On day one, animals were exposed to both the conditioned and neutral contexts for 15 minutes to acclimate. On day two, fish were trained to associate alarm substance exposure to the conditioned context. Conditioning trials consisted of three five minute blocks. For the first five minutes animals were allowed to acclimate to the arena. The second five minutes were recorded as an indicator of conditioned fear, and used to measure learning rate over four trials. Alarm substance (or DI water) was administered at the end of the conditioned fear block, and the fish’s unconditioned fear response was measured for five minutes. The conditioning trial was repeated four times with 30 minutes in their home tank between trials. On days three and seven, memory recall was tested by re-exposing fish to the conditioned and neutral contexts for 15 minutes each with two hours between contexts.

The paradigm consisted of three phases across 7 days of testing (Fig. 1): acclimation, conditioning, recall. Three days prior to testing, test subjects were moved from stock tanks into a behavioral testing room with individual housing to allow for individual identification throughout experiment. On day one (acclimation phase), fish were individually placed in the testing arena to acclimate for 15 minutes and then returned to their home tank. Two hours later this was repeated in the second context. On day two (conditioning phase), fish were conditioned to associate the white context with exposure to alarm substance over four conditioning trials. Each conditioning trial was 15 minutes long and was divided into three 5-minute blocks. Fish acclimated to the chamber for the first five minutes, followed by five minutes of recording the conditioned fear response. After these 10 minutes, 1 mL of alarm substance (or DI water in the control condition) was administered into the water through plastic tubing that came from outside of the testing arena. Following alarm substance exposure, the unconditioned fear response was recorded for five minutes. This procedure was repeated four times with 30 minute inter-trial intervals. Between trials, we placed fish back into their individual housing, rinsed out the testing arenas, and refilled with 1.4 L of fresh system water. On days three and seven (recall phase), animals were re-exposed to both the neutral context and the conditioned context for 15 minutes each, with two hours between tests. For acclimation and recall testing, the order of context exposure was counterbalanced across individuals. All behavioral testing was done between 2–6 hours after light onset and no sooner than 60 minutes following feeding. Further, all behavioral testing occurred within an opaque testing area with indirect fluorescent ceiling lighting and such that fish and experimenter had no direct visual access to each other during testing. All testing procedures were approved by the Institutional Animal Care and Use Committee of University of Nebraska at Omaha/University of Nebraska Medical Center (17-070-00-FC, 17-064-08-FC). All methods were performed in accordance with the relevant guidelines and regulations.

### Behavioral analysis

All trials were video-recorded from above and later analyzed with Noldus EthoVision XT (Noldus XT, Wageningen, Netherlands). For each trial, we quantified two measures as indicators of a conditioned response: freezing time and erratic movements. We examined these two behaviors because freezing is one of the most consistent and conserved behaviors used to assess stress-related behaviors and fear learning and memory^26,55^ and erratic movements are another ethologically relevant response to fear in teleost when exposed to alarm substance^29^.The subject was considered frozen if it moved less than 0.5 cm/s. Erratic movements were characterized as rapid darting and zig-zag movements. The duration of erratic movements was quantified using Ethovision’s Activity State analysis option (Noldus XT, Wageningen, Netherlands). The activity threshold was set to 99% and bins less than 0.1 seconds were removed. As erratic movements and freezing cannot occur simultaneously, we report duration of erratic movements as a proportion of total time spent moving. To validate software quantification of erratic movement duration, two independent observers manually recorded the duration of erratic movements for all of the unconditioned responses of the alarm substance group. Computer analyzed erratic movements were highly correlated with both observers (*r*_observer 1_ = 0.87, *p*_observer 1_ = 1.93 * 10^−15^ and *r*_observer 2_ = 0.91, *p*_observer 2_ = 2.77 * 10^−19^).

### Statistics

All statistics were performed using SPSS software (Version 24). For analysis of freezing and erratic movement durations on day one we used a repeated-measures two-way ANOVA with sex and strain as between-subject factors and conditioned vs. neutral as the within subjects factor. To assess whether there were any initial differences in behavior between treatment groups we also ran a three-way ANOVA with sex, strain, and treatment group as between-subject factors and conditioned vs. neutral as the within subjects factor. We conducted post-hoc individual comparisons with independent t-tests and applied the Benjamini-Hochberg correction to determine significance for all tests^56^.

For analysis of the learning phase (day 2), due to deviations from normality we used a repeated measures generalized estimating equation (GEE) with strain, sex, treatment group and trial (the four conditioning trials) as factors. As the behavior may be confounded by netting and handling, we ran analyses on the 2^nd^ five minutes of each conditioning trial (i.e. “conditioning fear response period”). To include behaviors displayed across the entire time in the arena prior to conditioning stimulus administration, we also analyzed the first 10 minutes of each conditioning trial. We conducted post-hoc pairwise comparisons with Mann-Whitney U tests. To examine changes in behavior across time within each conditioning trial, we ran separate repeated measures GEEs with strain, sex, and time (the two five minute blocks) as factors for the alarm substance treated fish and the control animals. We conducted post-hoc pairwise comparisons with Wilcoxon Signed Rank tests to examine directional effects of time on behavior. Given the documented relationship between body size and boldness, we attempted to control for this by entering standard length into the models as a covariate^48,57–59^. SPSS p-value outputs of “0” are considered as 1E-17. To account for multiple comparisons, we applied the Benjamini-Hochberg correction to determine significance for all tests^56^.

During the memory recall at days three and seven, we used a repeated-measures three-way ANOVA with sex, strain, and treatment group as between-subject factors and with conditioned vs. neutral context as the within subjects factor. We conducted post-hoc individual comparisons with independent t-tests and adjusted for multiple comparisons using Benjamini-Hochberg correction^56^. For significant differences (p < 0.05) in all of the above analyses we also report the effect sizes (Cohen’s d (d) for t-tests, Mann-Whitney U, and Wilcoxon Signed Rank tests; eta-squared (η^2^) and partial eta-squared (ηp^2^) for GEEs and ANOVAs, respectively)^60^. All effect sizes were medium or large effects^60–62^.

## Results

During Day 1 acclimation there were no significant within-subjects effects of context or any interaction effect on baseline freezing or erratic movement behaviors. There was a significant between-subjects main effect of strain on freezing time where HSB fish froze significantly more than LSB fish overall (2-Way ANOVA: *F*_1, 55_ = 7.51, p = 0.008, ηp^2^ = 0.11; 3-Way ANOVA: *F*_1, 55_ = 10.81, *p* = 0.002, ηp^2^ = 0.16). However, there were no other significant between-subjects effects or interaction effects for freezing, nor any for erratic movements (all *p* > 0.05; Fig. S2).

**Figure 2:**
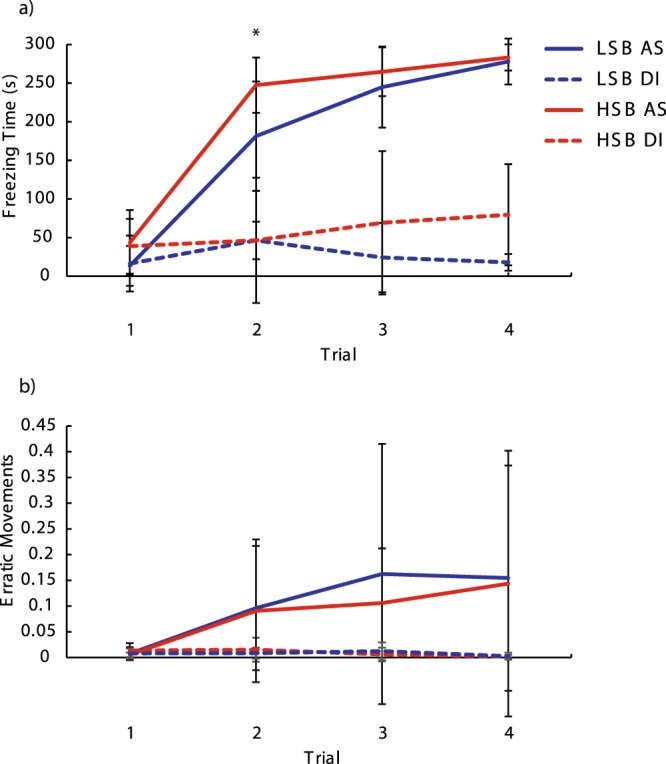
Acquisition of fear memory over four conditioning trials. Freezing time (**a**) and erratic movement ratio (**b**) were measured for high stationary behavior (HSB) and low stationary behavior (LSB) fish exposed to distilled water (DI) or alarm substance (AS). Points represent mean ± 1 standard deviation. *Indicates *p* < 0.05 for within-treatment group comparison.

During the conditioning phase (Day 2), fish that received alarm substance showed a significantly higher unconditioned response (five minute period post-administration) for freezing (*F*_1, 55_ = 563.41, *p* = 1.41 * 10^−30^, ηp^2^ = 0.91) and erratic movements (*F*_1, 55_ = 11.77, *p* = 0.001, ηp^2^ = 0.18) compared to DI water (Fig. S3). There were no other significant between-subjects effects or interaction effects for the unconditioned fear response (all *p* > 0.05). Within the conditioned fear response period (second five minute block), there were significant main effects of strain (Wald Chi-Square = 18.8, p = 1.5 * 10^−5^, η^2^ = 0.29), sex (Wald Chi-Square = 17.538, p = 2.8 * 10^−5^, η^2^ = 0.27), treatment (Wald Chi-Square = 502.15, p = 1.0 * 10^−17^, η^2^ = 1), and trial (Wald Chi-Square = 595.565, p = 1.0 * 10^−17^, η^2^ = 1) on freezing time. There was a significant trial * treatment interaction effect on both freezing (Wald Chi-Square = 420.404, p = 1.0 * 10^−13^, η^2^ = 1) and erratic movements (Wald Chi-Square = 57.838, p = 1.7 * 10^−12^, η^2^ = 1). The alarm substance group increased freezing across the four trials while the DI control group did not. Of note, there was also a significant trial * strain * treatment group interaction effect on freezing time (Wald Chi-Square = 8.553, p = 0.036, η^2^ = 0.13) where treated HSB fish increased freezing behavior at a faster rate than LSB fish (Fig. 2, Table S1). This significant strain * trial interaction effect remains when we only analyze alarm-substance treated fish (Wald Chi-Square = 10.03, p = 0.018, η^2^ = 0.21). HSB fish exposed to alarm substance froze significantly more than LSB fish at trial two (U = 145.4, *p* = 0.003, d = 0.937) and was not significant at trials one (U = 221, *p* = 0.165), three (U = 211.5, *p* = 0.114), or four (U = 251.5, *p* = 0.439) (Fig. 2). For erratic movement duration, there were only significant main effects of treatment (Wald Chi-Square = 49.023, p = 2.5 * 10^−12^, η^2^ = 0.77) and trial (Wald Chi-Square = 53.209, p = 1.65 * 10^−11^, η^2^ = 0.83). There was not a significant trial * strain * treatment group interaction effect for erratic movements (Wald Chi-Square = 1.474, p = 0.688). Full model results are presented in Table S1.

Expanding the analysis period to the first 10 minutes of each conditioning trial (first and second five minute blocks), we similarly see significant main effects of strain (Wald Chi-Square = 75.734, p = 1.0 * 10^−17^, η^2^ = 1), sex (Wald Chi-Square = 22.791, p = 1.8 * 10^−6^, η^2^ = 0.35), treatment (Wald Chi-Square = 1084, p = 1.0 * 10^−17^, η^2^ = 1), and trial (Wald Chi-Square = 1298, p = 1.0 * 10^−17^, η^2^ = 1), and significant trial * strain * treatment interaction effect (Wald Chi-Square = 10.489, p = 0.015, η^2^ = 0.16) on freezing time (Table S1). HSB fish exposed to alarm substance froze significantly more than LSB fish in the first three trials (Trial 1: U = 66, *p* = 4.7 * 10^−6^ d = 1.76; Trial 2: U = 57, *p* = 1.9 * 10^−6^ d = 1.89; Trial 3: U = 177, *p* = 0.022 d = 0.7) but not on trial four (U = 217, *p* = 0.143). We also see significant main effects of treatment (Wald Chi-Square = 95.575, p = 1.0 * 10^−17^, η^2^ = 1) and trial (Wald Chi-Square = 106.438, p = 1.0 * 10^−17^, η^2^ = 1) on erratic movements (Table S1). Full model results are presented in Table S1.

**Figure 3:**
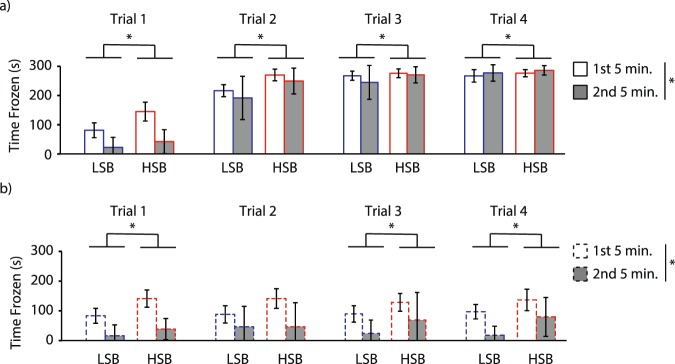
Habituation of freezing behavior across conditioning trials. We measured freezing time for (**a**) individuals exposed to alarm substance and (**b**) distilled water for the high stationary behavior (HSB) and low stationary behavior (LSB) strains over time. White and gray bars represent the 1^st^ and 2^nd^ five-minute blocks prior to stimulus administration, respectively. Bars represent mean ± 1 standard deviation. *Indicates *p* < 0.05.

Examining habituation within each conditioning trial on Day 2, there were significant main effects of strain and time (first and second five minute blocks) for all four trials in the alarm substance treated groups (Fig. 3, Table S2). The HSB fish showed significantly more time frozen than the LSB fish in all trials (Table S2). All fish decreased the amount of freezing time in the conditioned response period (second 5 minute block) relative to the acclimation period (first 5 minute block) for the first two conditioning trials (Trial 1: Z = 5.939, p = 2.8 * 10^−9^, d = 3.33; Trial 2: Z = 2.318, p = 0.02, d = 0.71) but not the third trial (Trial 3: Z = 1.815, p = 0.069). In trial four the amount of freezing time increased (Trial 4: Z = 2.923, p = 0.003, d = 0.93; Fig. 3). The DI treated individuals showed a significant main effect of time on freezing duration for all conditioning trials (Table S2) where fish decreased freezing time in the second five-minute block (Trial 1: Z = 3.464, p = 0.001, d = 3.46; Trial 2: Z = 2.482, p = 0.013, d = 1.58; Trial 3: Z = 2.534, p = 0.011, d = 1.64; Trial 4: Z = 3.103, p = 0.002, d = 2.46; Fig. 3). The main effect of strain was only seen in trials 1, 3, 4 and the main effect of sex was seen in trials 1,2, and 4 where the HSB and females spent more time frozen relative to LSB and males, respectively (Fig. 3, Table S2). There was a significant strain*time interaction effect on freezing time for only trial 1 in both treatment groups where HSB fish showed a greater reduction in freezing time in the unconditioned response period (second five minute block) relative to the acclimation period (first five minute block) (Table S2). Full model results are presented in Table S2.

**Figure 4:**
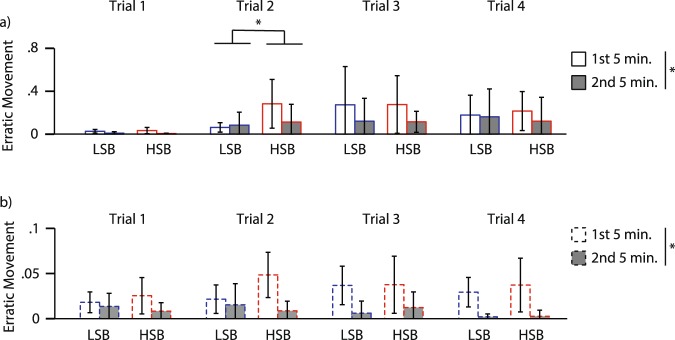
Habituation of erratic movement across conditioning trials. We measured erratic movement duration ratio for (**a**) individuals exposed to alarm substance and (**b**) distilled water for the high stationary behavior (HSB) and low stationary behavior (LSB) strains over time. White and gray bars represent the 1^st^ and 2^nd^ five-minute blocks prior to stimulus administration, respectively. Bars represent mean ± 1 standard deviation. *Indicates *p* < 0.05.

For erratic movements, there was a significant main effect of time for the first three conditioning trials but not the last one in the alarm substance treated group (Fig. 4, Table S3). Fish in these trials showed less amount of erratic movements in the second five minute block relative to the first block (Trial 1: Z = 4.882, p = 1 * 10^−6^, d = 1.99; Trial 2: Z = 2.349, p = 0.019, d = 0.72; Trial 3: Z = 3.282, p = 0.001, d = 1.08; Trial 4: Z = 1.767, p = 0.077; Fig. 4). There was only a significant main effect of strain and strain*time interaction effect on erratic movements in trial 2 (Table S3). DI treated individuals showed significant main effect of time on all conditioning trials (Table S3) where there was a decrease in amount of erratic movements in the second five minute block (Trial 1: Z = 1.913, p = 0.056, d = 1.09; Trial 2: Z = 2.792, p = 0.005, d = 1.95; Trial 3: Z = 3.103, p = 0.002, d = 2.46; Trial 4: Z = 3.309, p = 0.001, d = 2.95; Fig. 4). Full model results are presented in Table S3.

**Figure 5:**
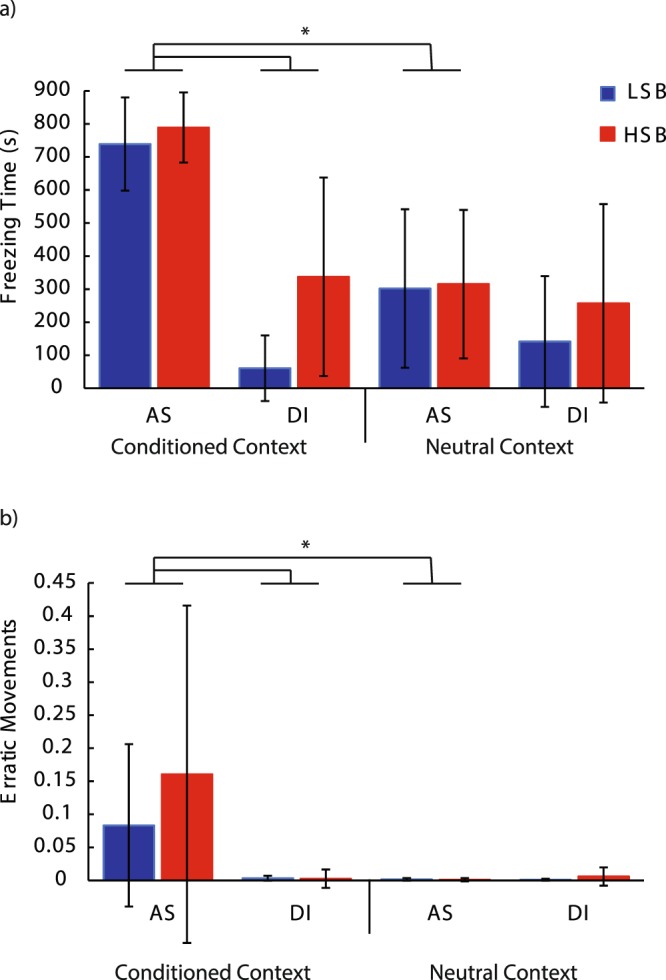
Fear memory recall 24 hours post-conditioning. We measured freezing time (**a**) and erratic movement ratio (**b**) for high stationary behavior (HSB) and low stationary behavior (LSB) fish exposed to distilled water (DI) or alarm substance (AS) during conditioning. Bars represent mean ± 1 standard deviation in the conditioned context and neutral context. *Indicates *p* < 0.05.

**Figure 6:**
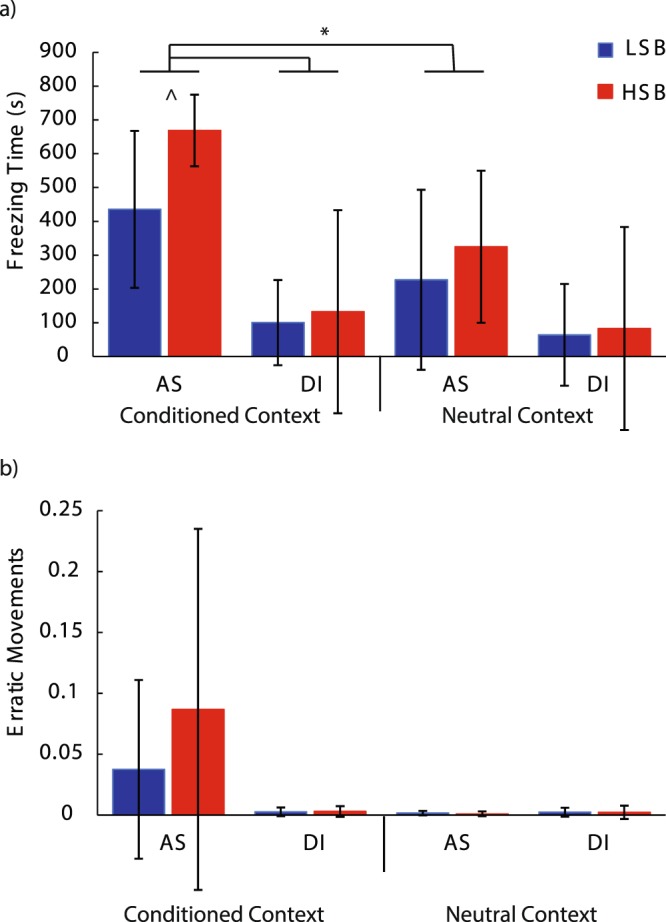
Fear memory recall 96 hours post-conditioning. We measured freezing time (**a**) and erratic movement ratio (**b**) for high stationary behavior (HSB) and low stationary behavior (LSB) fish exposed to distilled water (DI) or alarm substance (AS) during conditioning. Bars represent mean ± 1 standard deviation in the conditioned context and neutral context. *Indicates *p* < 0.05. ^^^Indicates p < 0.05 for within-treatment group comparison in the conditioned context.

During memory recall testing there was a significant context * treatment group interaction effect for both behaviors at 24 h (Freezing: *F*_1, 55_ = 49.45, *p* = 2.97 * 10^−9^, ηp^2^ = 0.48, erratic movements: *F*_1, 55_ = 5.41, *p* = 0.024, ηp^2^ = 0.09, Fig. 5) and freezing behavior at 96 h (*F*_1, 55_ = 8.03, *p* = 0.006, ηp^2^ = 0.127, Fig. 6) post-conditioning. In the alarm substance, but not the DI water group, both strains displayed significantly higher antipredatory behaviors in the conditioned context compared to the neutral context. At 96 hours post-conditioning, there was a significant strain*treatment interaction effect for freezing behavior (*F*_1, 55_ = 4.13, *p* = 0.047, ηp^2^ = 0.07). Treated HSB fish showed significantly higher freezing behavior compared to treated LSB fish in the conditioned context at 96 h (*t*(46) = 3.62, *p* = 0.001, d = 1.01), meanwhile DI water treated animals showed similar basal levels of freezing behavior in both contexts. Full model results are presented in Table S4.

## Discussion

While it is essential for animals to encode and recall salient experiences, it is unclear how different stress coping styles may influence the use of contextual information to predict and avoid danger in the future. In the present study, we measured the learning rate and duration of a fear memory in selectively-bred lines of zebrafish that on average display proactive and reactive coping styles. Overall, we found that zebrafish from the reactive strain (HSB) acquired the association of a fearful olfactory stimulus with contextual information more quickly and retained this fear memory longer compared to those from the proactive strain (LSB).

Learning rate and memory duration can differ amongst individuals with different personality types^7,12^. During conditioning, despite showing no significant difference in freezing at the start (trial 1), reactive zebrafish (HSB strain) showed significantly higher amounts of freezing (trial 2) compared to proactive individuals (LSB strain). By the end of conditioning (trial 4), there was no significant difference in freezing time between the strains when examining the conditioning block (2^nd^ five minutes). This suggests that reactive zebrafish acquire a contextual fear memory at a significantly faster rate than proactive zebrafish. It should be noted that the proactive zebrafish will eventually display the same amount of freezing as the reactive zebrafish but require at least one more re-exposition to alarm substance. While intratrial habituation is clearly occurring, there is no significant strain*time interaction effect on freezing or erratic movements within any conditioning trial for fish exposed to alarm substance. Therefore, it is unlikely that a potentially faster rate of non-associative learning by the proactive fish could explain our results. We also do not have evidence to support differences in alarm response thresholds influencing our results as there were no significant strain differences in freezing and erratic behaviors after first exposure to the alarm substance (unconditioned fear response period during first conditioning trial). When analyzing both the acclimation and conditioning blocks (first 10 minutes of trial), we observed overall similar results but with stronger effect sizes. In particular, the reactive zebrafish spent significantly higher amounts of time frozen than the proactive over the first three conditioning trials and with larger effect sizes. As fish exposed to either alarm substance or distilled water showed habituation in freezing behavior and erratic movements over all conditioning trials, it suggests that the netting and handling process is contributing to these stronger responses observed when analyzing the first 10 minutes.

Faster learning rates in reactive individuals have also been observed in other teleost^15,23^ and avian species^16,17^. With higher tendencies to exhibit risk-averse behaviors and elevated cortisol responses, we hypothesize that reactive individuals may perceive stressors as more threatening, which could facilitate faster encoding of aversive experiences. While studies have documented faster learning proactive individuals^18–22,24^, this may be due to different learning tasks or type of reinforcing stimulus. Reactive individuals have higher learning performance with aversive conditioning whereas proactive individuals tend to learn more quickly in exploratory or discrimination tasks with appetitive conditioning^15,19–21,23^. The current study only examined two commonly utilized behavioral responses to fear and used one ecologically relevant stimulus to induce fear. While freezing behavior shows strong consistent within and between individual differences for both the proactive and reactive zebrafish strains used in this study^52^, congruency with other non-locomotor based endpoints (e.g. sympathetic responses) would provide additional support for strain differences in learning. Additional studies utilizing other stimuli and paradigms are also needed to assess if the effects observed here are paradigm-specific.

Freezing time and erratic movements during the recall phase indicated that both strains recalled the fear memory at least four days following conditioning. However, the HSB fish showed significantly higher levels of freezing in the conditioned context at 96 hours suggesting that reactive individuals encode a more resilient fear memory than proactive individuals (Fig. 4). Differences in learning and memory between stress coping styles are seen in both contextual (e.g. general environment) and cued (e.g. specific neutral odors or visual stimuli) learning of salient information using a threatening stimulus. Animals displaying a reactive coping style may repress exploratory behavior and be more risk-averse for longer when re-exposed to potentially dangerous contexts or cues to minimize risks of injury. This interpretation is consistent with other studies suggesting that reactive individuals retain fearful memories for longer^10,16^. However, one study found that proactive rainbow trout retained a conditioned fear response for longer, which may be due to the reactive trout having faster extinction learning^25^, or because a physiological measure (cortisol) was used as a conditioned fear response as opposed to behavior. One potential confound of the recall results is the inability to separate out effects of memory reconsolidation or memory extinction. Animals were re-exposed to the conditioned context twice following the conditioning day but without alarm substance and the first recall trial may have differential consequences between the strains. More specifically, we cannot rule out the possibility that the proactive strain may be exhibiting faster extinction learning or longer-term non-associative learning via habituation. A more thorough longitudinal study where animals are tested at just one time point during recall will help minimize these impacts.

Painful or frightening stimuli can quickly modify current and future behavioral responses. Studies using electric shocks in fear conditioning have revealed important insights into the proximate mechanisms of learning and memory^26,55^. However, electric shocks have limited ecological relevance to the evolution of adaptive animal behavior. Predator odors or chemical alarm signals are alternative, but ecologically relevant aversive conditioning stimuli^63,64^. While alarm substance is used as an aversive conditioning stimulus in other studies utilizing teleosts^10,30–34,65^, our conditioning paradigm allows for effective analysis of behavior at the individual level and achieved an unconditioned response rate in ~75% of fish. Further, alarm substance induced similar unconditioned fear responses in both proactive and reactive zebrafish. Only fish exposed to alarm substance displayed increasing conditioned fear responses across learning trials and presented high levels of freezing in the conditioned context during memory recall. This is consistent with freezing and avoidance behaviors observed in other fear conditioning paradigms utilizing chemical alarm signals and electric shocks^27,32,66^. One potential limitation of this paradigm is the necessity to individually house animals for the duration of the experiment to allow for tracking. Prior studies document behavioral and biochemical alterations due to chronic social isolation (>90 days)^67,68^. However, this paradigm only requires zebrafish to be isolated for 10 days and animals can still visually and chemically detect each other. Therefore we predict impacts of isolation will be minimal. Additionally, we’ve shown that zebrafish physically isolated in the same manner showed no significant changes in stress-related behaviors over 5 weeks of testing in either the HSB or LSB strains^52^. It is noteworthy that our paradigms used colored chambers as the conditioned and neutral contexts and that zebrafish can have innate preferences for specific colors^69^. While the HSB and LSB strains showed no significant differences in freezing or erratic movements during Day 1 acclimation between the conditioned or neutral context for either colors, which suggests no innate preferences for colors used here, color biases should be assessed in other strains prior to selecting colors. Collectively our paradigm can be used to measure contextual learning and memory in individual zebrafish as fish acquired the association between the alarm substance and the contextual information, and were able to discriminate between the conditioned and neutral contexts.

In summary, we document several interaction effects between an individual’s stress coping style and learning and memory of a fearful association. Despite showing similar acute responses to potential predation, we find that contextual fear learning rates differ by our strains representing the reactive and proactive stress coping styles. Specifically, reactive individuals showed a faster learning rate than proactive individuals, which cannot be explained by differential habituation. We also observed differences in behavior between strains when tested four days post-conditioning where the data shows reactive individuals having a greater response. This could be due to reactive individuals having a longer memory but potential extinction learning differences need to be ruled out. The differences in learning and memory performances between the strains may be due to different molecular priming of synaptic plasticity and neurotransmission related genes in the brain^41^. We also show that alarm substance and our paradigm can be used to understand contextual learning and memory differences at the individual level. It is important to consider a variety of paradigms as different associations and reinforcement valences may incur different sets of tradeoffs that influence cognition. Lastly, these behavioral findings present a promising basis to investigate the neuromolecular mechanisms underlying cognitive biases and stress coping styles.

## Supplementary information

Supplementary Information

Dataset 1

## Data Availability

All data generated or analyzed during this study are included in this published article and its Supplementary Information files.
