# Supplementary material for: Contextual fear learning and memory differ between stress coping styles in zebrafish
Source: Sci Rep. 2019 Jul 9;9:9935. doi: 10.1038/s41598-019-46319-0 (PMC6617452; doi:10.1038/s41598-019-46319-0)
Supplement: Supplementary file 1 — Supplementary Information [file 41598_2019_46319_MOESM1_ESM.pdf]

Supplementary Information

Contextual fear learning and memory differ between stress coping styles in zebrafish

Matthew R Baker[1] and Ryan Y Wong[1, 2,*]

[1]Department of Biology, University of Nebraska at Omaha, Omaha, Nebraska, USA

[2]Department of Psychology, University of Nebraska at Omaha, Omaha, Nebraska, USA

*Correspondence: Ryan Y Wong, University of Nebraska at Omaha, 6001 Dodge St, Omaha, NE 68182 Email: rwong@unomaha.edu Phone: 402-554-4473

Tables

Table S1. Results of repeated measures GEE for the acquisition learning phase for freezing time and erratic movement ratio.

	**Freezing Time**		**Erratic Movement**	
	Conditioning Period (2nd five minute block)	Acclimation & Conditioning Periods (1st and 2nd five minute blocks)	Conditioning Period (2nd five minute block)	Acclimation & Conditioning Periods (1st and 2nd five minute blocks)
**Effects**	Wald χ^2 (p-value, η^2)	Wald χ^2 (p-value, η^2)	Wald χ^2 (p-value, η^2)	Wald χ^2 (p-value, η^2)
Intercept	**1195.355 (1E-17, 1)**	**5070.867 (1E-17, 1)**	**71.659 (1E-17, 1)**	**180.279 (1E-17, 1)**
Strain	**18.800 (1.4E-5, .29)**	**75.734 (1E-17, 1)**	0.077 (.781)	0.464 (.496)
Sex	**17.538 (2.8E-5, .27)**	**22.791 (1.8E-6, .35)**	0.564 (.453)	0.007 (.934)
Treatment	**502.150 (1E-17, 1)**	**1084.015 (1E-17, 1)**	**49.023 (2.5E-12, .77)**	**95.575 (1E-17, 1)**
Trial	**595.565 (1E-17, 1)**	**1298.690 (1E-17, 1)**	**53.209 (1.6E-11, .83)**	**106.438 (1E-17, 1)**
Strain * Sex	0.672 (.412)	0.156 (.693)	**4.273 (.039, .07)**	0.006 (.938)
Strain * Treatment	0.095 (.758)	1.213 (.271)	0.028 (.868)	0.150(.699)
Strain * Trial	1.616 (.656)	2.187 (.534)	0.928 (.819)	5.459 (.141)
Sex * Treatment	**13.158 (2.8E-4, .21)**	**21.050 (4.4E-6, .33)**	0.002 (.967)	0.282 (.596)
Sex * Trial	2.411 (.492)	1.123 (.771)	2.187 (.535)	0.392 (.942)
Treatment * Trial	**420.404 (1E-17, 1)**	**982.819 (1E-17, 1)**	**57.838 (1.7E-12, 1)**	**92.853 (1E-17, 1)**
Strain * Sex * Treatment	**4.851 (.028, .08)**	**5.326 (.021, .08)**	3.805 (.051)	0.049 (.825)
Strain * Sex * Trial	3.259 (.353)	1.410 (.703)	9.327 (.025,)	5.331 (.149)
Strain * Treatment * Trial	**8.553 (.036, .13)**	**10.489 (.015, .16)**	1.474 (.688)	2.911 (.405)
Sex * Treatment * Trial	3.073 (.38)	3.251 (.354)	3.596 (.308)	0.230 (.973)
Strain * Sex * Treatment * Trial	3.110 (.375)	1.203 (.752)	**10.745 (.013, .17)**	6.848 (.077)

Bold text indicates $p < 0.05$.

Table S2. Results of repeated measures GEE for analysis of changes in behavior between first and second five minute periods within each conditioning trial for freezing time.

	Trial 1	Trial 2	Trial 3	Trial 4
	Wald χ^2 (p-value, η^2)	Wald χ^2 (p-value, η^2)	Wald χ^2 (p-value, η^2)	Wald χ^2 (p-value, η^2)
**Effects (Alarm Substance Treated)**				
Intercept	**449.170 (1E-17, 1)**	**2513.154 (1E-17, 1)**	**5284.181 (1E-17, 1)**	**14866.446 (1E-17, 1)**
Strain	**36.955 (1.2E-9, .77)**	**36.364 (1.6E-9, .76)**	**5.511 (.019, .11)**	**3.901 (.048, .08)**
Sex	1.972 (.16)	0.020 (.886)	0.469 (.494)	0.004 (.95)
Time	**164.334 (1E-17, 1)**	**6.667 (.01, .14)**	**5.193 (.023, .11)**	**7.851 (.005, .16)**
Strain * Time	12.455 (4.1E-4,)	0.059 (.808)	1.985 (.159)	0.002 (.967)
Sex * Time	2.924 (.087)	0.423 (.516)	0.050 (.823)	0.545 (.46)
**Effects (DI Treated)**				
Intercept	**231.612 (1E-17, 1)**	**104.170 (1E-17, 1)**	**76.454 (1E-17, 1)**	**185.194 (1E-17, 1)**
Strain	**19.036 (1.2E-5, .59)**	2.840 (.092)	**5.554 (.018, .17)**	**17.345 (3.1E-5, .54)**
Sex	**13.218 (2.7E-4, .41)**	**12.261 (4.6E-4, .38)**	3.092 (.079)	**6.991 (.008, .22)**
Time	**112.511 (1E-17, 1)**	**21.532 (3.4E-6, .67)**	**14.605 (1.3E-4, .46)**	**26.627 (2.4E-7, .83)**
Strain * Time	**4.915 (.027, .15)**	3.243 (.072)	0.035 (.851)	0.695 (.404)
Sex * Time	0.895 (.344)	**4.919(.027, .15)**	1.552 (.213)	0.116 (.734)

Bold text indicates $p < 0.05$.

Table S3. Results of repeated measures GEE for analysis of changes in behavior between first and second five minute periods within each conditioning trial for erratic movement ratio.

	Trial 1	Trial 2	Trial 3	Trial 4
	Wald χ^2 (p-value, η^2)	Wald χ^2 (p-value, η^2)	Wald χ^2 (p-value, η^2)	Wald χ^2 (p-value, η^2)
**Effects (Alarm Substance Treated)**				
Intercept	**108.657 (1E-17, 1)**	**63.925 (1.3E-17, 1)**	**66.724 (3.3E-17, 1)**	**68.852 (1.1E-17, 1)**
Strain	0.144 (.705)	**13.530 (2E-4, .28)**	0.001 (.97)	0.002 (.968)
Sex	0.001 (.979)	0.175 (.676)	0.000 (.994)	0.693 (.405)
Time	**38.749 (4.8E-10, .81)**	**7.532 (.006, .16)**	**9.000 (.003, .19)**	1.557 (.212)
Strain * Time	2.000 (.157)	**12.295 (4.5E-4, .26)**	0.006 (.938)	0.775 (.379)
Sex * Time	1.455 (.228)	0.530 (.466)	0.189 (.664)	1.406 (.236)
**Effects (DI Treated)**				
Intercept	**64.648 (8.8E-16, 1)**	**48.420 (3.4E-12, 1)**	**33.568 (6.8E-9, 1)**	**43.959 (3.3E-11, 1)**
Strain	0.056 (.813)	2.223 (.136)	0.191 (.662)	0.617 (.432)
Sex	0.081 (.776)	0.796 (.372)	1.850 (.174)	0.156 (.693)
Time	**4.067 (.044, .13)**	**16.558 (4.7E-5, .52)**	**24.046 (9.4E-7, .75)**	**26.092 (3.2E-7, .82)**
Strain * Time	1.375 (.241)	**8.830 (.003, .28)**	0.227 (.634)	0.360 (.548)
Sex * Time	0.140 (.709)	2.069 (.15)	0.673 (.412)	0.152 (.697)

Bold text indicates $p < 0.05$.

Table S4. Results of repeated measures GLM for the memory recall phase for freezing time and erratic movement ratio at 24h and 96h post training.

	**51.15 (2.75E-9, .48)**24h Freezing Time	24h Erratic Movement	96h Freezing Time	96h Erratic Movement
	F(p, ηp^2)	F(p, ηp^2)	F(p, ηp^2)	F(p, ηp^2)
Within-Subjects Effects (df = 1, 55)				
Context	1.21 (.277)	0.02 (.900)	3.31 (.074)	0.10 (.755)
Context*Strain	1.82 (.518)	0.63 (.430)	0.10 (.754)	0.79 (.378)
Context*Sex	0.06 (.805)	0.09 (.762)	0.94 (.336)	0.42 (.521)
Context*Treatment	**49.45 (2.97E-9 .48)**	**5.41 (.024, .09)**	**8.03 (.007, .13)**	3.54 (.065)
Context*Strain*Sex	0.83 (.365)	0.02 (.900)	0.82 (.370)	0.00 (.963)
Context*Strain*Treatment	1.04 (.312)	0.82 (.369)	0.12 (.726)	0.68 (.413)
Context*Sex*Treatment	0.89 (.351)	0.05 (.823)	1.79 (.187)	0.67 (.415)
Context*Strain*Sex*Treatment	0.52 (.472)	0.01 (.946)	0.22 (.645)	0.03 (.862)
Between Subjects Effects (df = 1, 55)				
Intercept	0.07 (.791)	0.01 (.928)	0.32 (.572)	0.12 (.735)
Strain	7.17* (.010, .03)	0.78 (.382)	**7.60 (.009, .12)**	0.24 (.630)
Sex	0.49 (.488)	0.06 (.802)	0.26 (.613)	0.76 (.387)
Treatment	**51.31 (1.97E-9, .483)**	**4.99 (.030, .08)**	**51.15 (2.75E-9, .48)**	3.27 (.076)
Strain*Sex	1.52 (.223)	0.00 (.998)	1.77 (.188)	0.02 (.899)
Strain*Treatment	3.47 (.068)	0.65 (.425)	**4.13 (.047, .07)**	0.65 (.423)
Sex*Treatment	**6.65 (.013, .11)**	0.12 (.727)	1.11 (.296)	0.65 (.424)
Strain*Sex*Treatment	**4.33 (.045, .07)**	0.04 (.845)	0.01 (.909)	0.00 (.989)

Bold text indicates $p < 0.05$

Figures

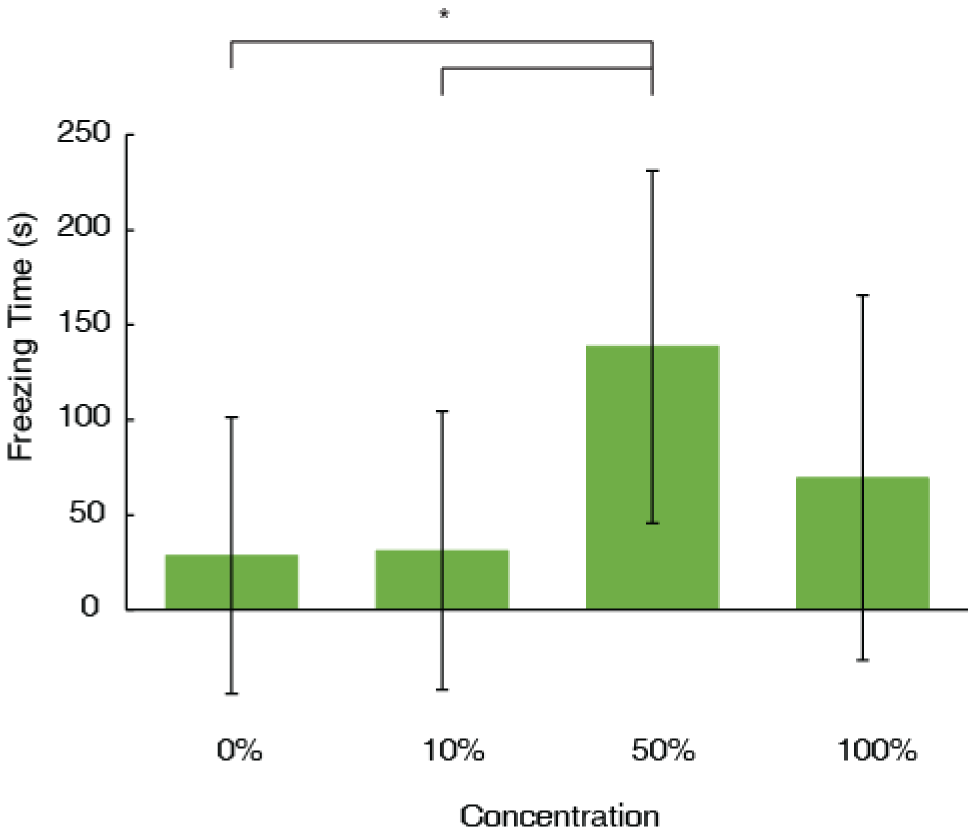

Figure S1. Dose response analysis of alarm substance administration on freezing behavior. For pilot trials, fish were recorded for five minutes after administration of four concentrations of alarm substance (DI water, 10%, 50%, 100%). Bars indicate mean ± 1 standard deviation. * indicates $p < .05$.

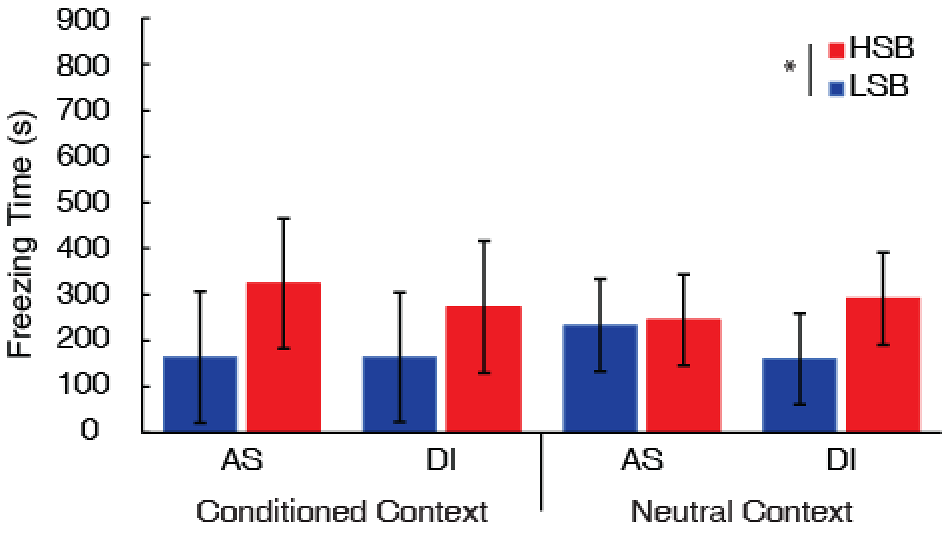

Figure S2. Freezing time displayed during acclimation phase. We measured freezing time for high stationary behavior (HSB) and low stationary behavior (LSB) fish exposed to distilled water (DI) or alarm substance (AS). Bars represent mean ± 1 standard error in the conditioned context and neutral context. Overall, HSB fish froze significantly more than LSB fish. However, there was no effect of context or treatment group on freezing time.

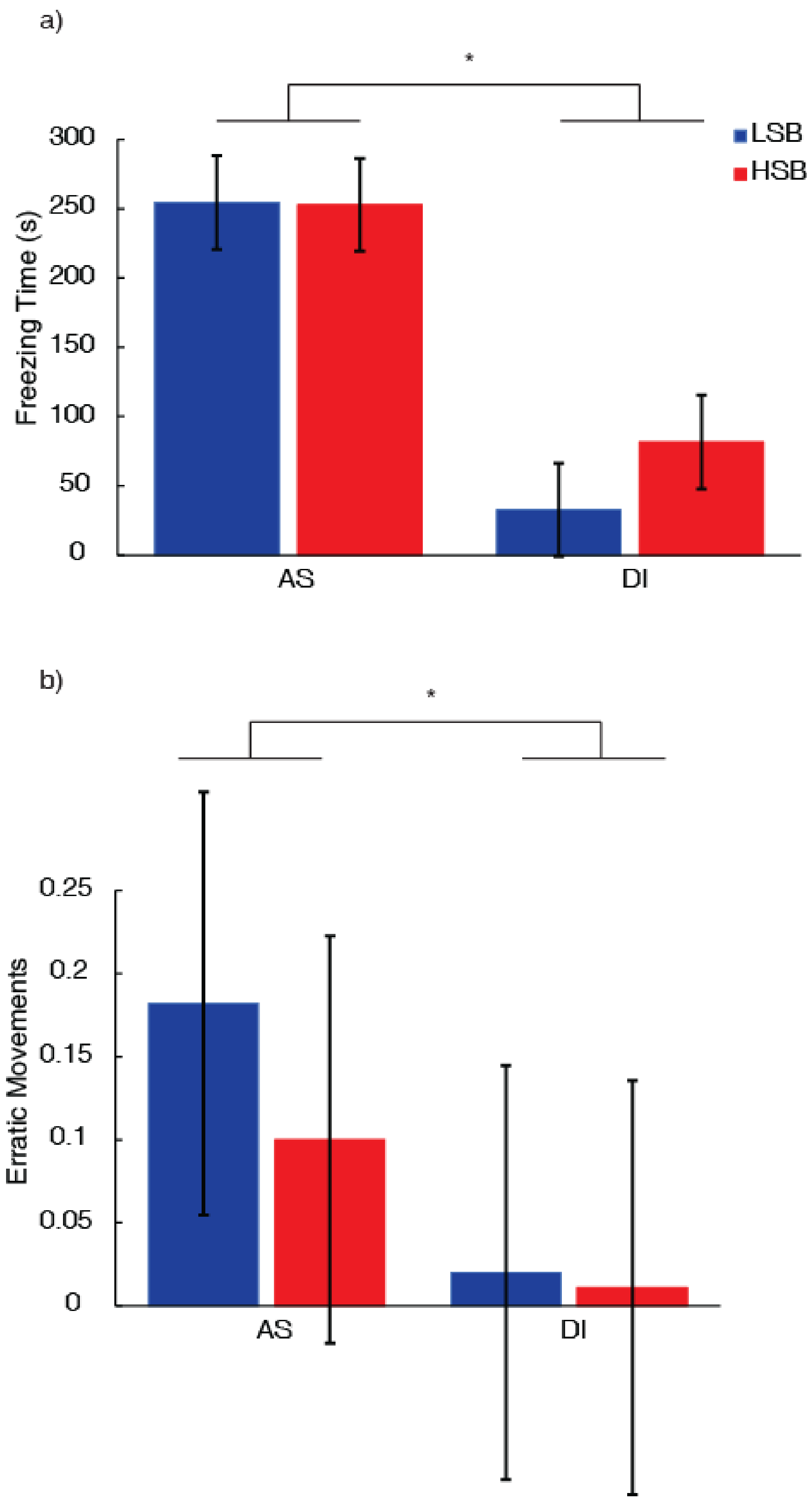

Figure S3. Unconditioned fear response during the first learning trial. We measured freezing time (A) and erratic movement ratio (B) for high stationary behavior (HSB) and low stationary behavior (LSB) fish exposed to distilled water (DI) or alarm substance (AS). Bars represent mean ± 1 standard deviation in the conditioned context. * indicates $p < .05$.
